# National Cancer Control Programme in India: Proposal for Organization of Chemotherapy and Systemic Therapy Services

**DOI:** 10.1200/JGO.2015.001818

**Published:** 2016-06-22

**Authors:** Seema Gulia, Manju Sengar, Rajendra Badwe, Sudeep Gupta

**Affiliations:** All authors, Tata Memorial Centre, Parel, Mumbai, India.

## Abstract

Cancer is a major health problem in India, with an estimated incidence of 1 million cases in 2012 that is likely to double in 2035 to approximately 1.7 million. The majority of cases are diagnosed in advanced stages, and approximately two thirds of patients die as a result of their disease. The mortality-to-incidence ratio is 0.68 in India, which is far higher than that in developed countries (approximately 0.38). One of the important reasons for this discrepancy is inequitable distribution and inaccessibility of health care resources in India. One component of scarce health care resources is the low ratio of oncologists to patients with cancer (1:2,000), which leads to delivery of systemic anticancer therapy in many hospitals by health care professionals who do not have required training. Given these facts, there is a need to focus on organization of medical oncology services in terms of manpower and infrastructure to standardize the delivery of systemic anticancer therapy. Redistribution of resources can streamline the delivery of cancer care, preferably close to the patient’s home. This article describes the blueprint for organization of medical oncology services and delivery of chemotherapy and other systemic therapies to Indian patients. The model uses existing health care services in the country and is a four-tiered system of increasing sophistication: District Hospitals, Medical College Hospitals, Regional Cancer Centres, and Apex Cancer Centres. Delivery of quality care to patients with cancer through standardized protocols is crucial in improving cancer outcomes in India.

## INTRODUCTION

Cancer is a major health problem in India. According to GLOBOCAN estimates, cancer incidence in India was 1 million cases per year in 2012 and is likely to nearly double to 1.7 million by 2035.^1^ The majority of patients are diagnosed in advanced stages and approximately two thirds die as a result of their disease.^1^ The mortality-to-incidence ratio is 0.68 in India, which is far higher than that in developed countries (approximately 0.38).^2^ One of the important reasons for this differential is inequitable distribution and inaccessibility of health care resources in this country.^2^

Systemic therapy—either as primary, adjunct, or palliative treatment—is a key component of multidisciplinary management in the majority of cancers. Discovery of new drugs and better use of existing drugs has resulted in improved survival rates and/or quality of life in many cancers. The most common cancers in India requiring routine systemic therapy are those of breast, lung, gastric, colorectal, ovarian, and hematolymphoid origins. Together these cancers constitute approximately 0.4 million new cases per year.^1^ There are fewer than 1,000 formally trained medical oncologists in India, with the ratio of oncologists to patients with cancer being 1:2,000 compared with 1:100 in the United States.^3^ As expected, this leads to suboptimal delivery of systemic anticancer therapy to most patients by health care professionals who do not have requisite training.

Given these facts, there is a need to focus on organization of medical oncology services in terms of human resources and infrastructure to ensure optimal delivery of systemic anticancer therapy. Here it may be noted that although the age-standardized cancer incidence in India is approximately one fourth of that in the United States and other developed countries, the burden of cancer cases is approximately the same because of its larger population.

Many developed countries have well-organized medical oncology services and guidelines for safe and effective use of chemotherapy drugs in health care settings that are close to the patient’s place of residence.^4,5^ Being able to get effective treatment without having to undertake extensive travel improves patient compliance and outcomes.^6^ Thus, there is an urgent need to develop models for effective and safe delivery of systemic therapy (including chemotherapy) to eligible patients in India as one of the integral components of a well-rounded National Cancer Control Programme. The medium- and long-term deliverable would be reduction in cancer mortality. This article describes a blueprint for organization of medical oncology services and delivery of chemotherapy and other systemic therapies to Indian patients.

Our proposal for streamlined medical oncology services at the secondary and tertiary health care levels will aid in achievement of following objectives: (1) equitable access to standard treatment including chemotherapy and other systemic therapy; (2) delivery of safe and optimal systemic anticancer therapy close to the patient’s home through development of standard guidelines for procurement, storage, and administration of drugs and monitoring of outcomes; and (3) establishment of a hierarchical referral and training network of oncology services embedded within the existing multitiered public health system.

## METHODS

We performed an extensive literature search and a thorough review of the organization of chemotherapy services in various countries, and evaluated international guidelines and other reference documents as a reference point for our proposal.^2-5^

We carefully considered the existing public sector health care delivery mechanism in India, which is a four-tiered system of increasing sophistication^10^:

Primary Health Centres, located at the village level, comprise a six-bed hospital with one to two medical officers, three to four nursing staff, and one laboratory technician.^11^ Primary health services mainly provide outpatient services and limited inpatient care.
District Hospitals (secondary level) are located at the administrative headquarters of a geographically defined area, and number approximately 600 in India. District Hospitals provide outpatient, inpatient, and emergency services for a defined population. It is usually a 100- to 200- hospital with 30 to 35 medical officers, 70 to 80 nursing staff, and 15 to 20 technicians to perform laboratory and radiologic investigations. These hospitals provide consultation and inpatient services in general medicine, general surgery, obstetrics and gynecology, pediatrics, emergency care, critical care, anesthesia, ophthalmology, otolaryngology, dermatology, orthopedics, radiodiagnosis, dental care, and public health management.^11^ Thus, the multidisciplinary template to provide a service such as chemotherapy for common cancers exists in these hospitals.
Medical College Hospitals (tertiary level) number approximately 380, including 150 in the public sector. These are multispecialty centers with well-organized human resources, infrastructure, and postgraduate training facilities for basic departments such as internal medicine, general surgery, obstetrics and gynecology, ophthalmology, and so on, but mostly lack formal subspecialty departments such as oncology, cardiology, neurology, and others.
Apex Cancer Centres, which number approximately seven to eight, have high-level expertise, infrastructure, and training mandates for most subspecialty departments, including medical oncology.
In addition to the above, there is a separate oncology vertical of so-called Regional Cancer Centres, 27 in total, which are standalone cancer centers partly supported by public funding. These centers have trained surgical and radiation oncology personnel but are mostly lacking in formally trained medical oncology personnel.

We next considered the level of risk that various chemotherapy regimens pose in terms of probability of serious adverse effects as well as complexity of monitoring. In this context, it is recognized that not all cancers or cancer treatments are equivalent in terms of risk and complexity. Therefore, it is unlikely that all systemic therapy regimens could be safely delivered at all levels. Upon evaluation, we propose adoption of a risk-stratification scheme of chemotherapy regimens that accounts for patient-related factors and details of treatment protocol (Data Supplement).^5^

We then carefully considered the human resources and expertise available at each health care level in India and propose a model for safe delivery of chemotherapy at these levels (Data Supplement).^7-9^ Also suggested is an integrated organization of services at each level that we deem necessary for safe and effective delivery of chemotherapy (Data Supplement). Of note, this proposal mostly uses resources, with additional training when necessary, that are already available at each health care level in India.

We are also cognizant of the fact that existing human resources and infrastructure need to be augmented at all levels to achieve optimum results of cancer treatment, including chemotherapy. Therefore, keeping in mind the expected complexity and number of cancer cases at each level, we also propose human resource and infrastructure recommendations at each health care level that will guide policymakers in their decision making for allocation of resources (Data Supplement).

Last, a monitoring and quality-check mechanism needs to be an integral component of any new proposal for health care delivery. We therefore propose a framework for monitoring use and quality of chemotherapy delivery at various health care levels in India (Data Supplement). This framework is intended to assist in identification of the current capacity for safe service provision and to highlight areas for improvement. The framework will also help in evaluating feasibility and applicability of the proposed model.

## DISCUSSION

The burden of cancer in India is linked to inequities in health care access and uneven distribution of infrastructure and human resources across the country. Redistribution of resources can streamline delivery of cancer care, preferably close to the patient’s home. This is feasible with the development of close networking and links between District Hospitals, Medical College Hospitals, and Apex Cancer Centres. Delivery of quality care to patients with cancer through standardized protocols is crucial to improving cancer outcomes in India.

This proposal has been designed to facilitate safe and effective delivery of chemotherapy at various levels of health care delivery in India, starting at the secondary level of District Hospitals. Of note, we have consciously excluded the primary level from the ambit of therapeutic services because we believe that this will not be possible given existing human resource and infrastructure constraints. A defining feature of our proposal is that it uses the existing public sector health care delivery mechanism in India rather than create a separate oncology vertical. This is likely to be the most practical and feasible strategy in the long term.

We have proposed that delivery of low-risk chemotherapy at the District Hospital level be done under the supervision and responsibility of internal medicine specialists who are already available at these centers. Their previous training will enable these physicians to be quickly trained for this purpose. Although it would be ideal to make medical oncologists available at all such centers, this is unlikely to be possible in the foreseeable future for a variety of reasons, mainly the dearth of such specialists in India.

The mechanism proposed herein will require some investment in both human resources and infrastructure from the federal and state governments in India. Such an investment is likely to result not only in establishment of a nationwide chemotherapy/systemic therapy delivery mechanism, but also multidisciplinary care of patients with cancer. We acknowledge that, to some extent, establishing or improving the surgical and radiation therapy delivery mechanism will need to be undertaken concurrently, at least at the tertiary (Medical College Hospital) level. However, it is possible that the latter, being short-duration treatments, could be partly centralized in high-volume centers.

There are some weaknesses in our proposal. The main one is the fact that, although it has been rationally designed on the basis of models in existence elsewhere and the existing ground realities in India, there is lack of empirical evidence for its effectiveness. However, at present, there is no validated practice model in India for safe and effective administration of systemic therapy to patients with cancer in health care settings that are close to the patient’s place of residence. If our proposed framework (or a modification thereof) were to be implemented in the public health care system in India, an ongoing audit of feasibility and outcome monitoring on representative samples would be instructive as to its effectiveness as a delivery model.

With this in mind, we have also proposed metrics for ongoing evaluation of its implementation (Data Supplement). We acknowledge that it would be an iterative process to develop the final model. The long-term deliverable, which will require collaboration with the National Cancer Registry Programme, would be reduction in cancer mortality and morbidity. It is possible that health policy planners in India might want to consider this proposal for initial pilot implementation in a few geographically defined administrative units, such as some states or districts. Because most states in India already have the four-tiered health care mechanism in place, our proposal lends itself nicely to such pilot testing followed by modification based on initial results.

In conclusion, we propose herein a model for widespread delivery of chemotherapy in India by using the existing health care infrastructure. If found acceptable after pilot testing, this model could become an integral component of the National Cancer Control Programme of this country.^12^
